# Remote ischemic preconditioning preserves Connexin 43 phosphorylation in the rat heart *in vivo*

**DOI:** 10.1186/s12967-014-0228-8

**Published:** 2014-08-27

**Authors:** Timo Brandenburger, Ragnar Huhn, Andreas Galas, Benedikt H Pannen, Verena Keitel, Franziska Barthel, Inge Bauer, André Heinen

**Affiliations:** Department of Anesthesiology, University Hospital Duesseldorf, Moorenstr. 5, 40225 Düsseldorf, Germany; Clinic for Gastroenterology, Hepatology and Infectious Diseases, Heinrich-Heine-University, 40225 Düsseldorf, Germany; Department of Cardiovascular Physiology, Heinrich-Heine-University, 40225 Düsseldorf, Germany

**Keywords:** Cardioprotection, Connexin 43 (Cx43), Remote ischemic preconditioning (RIPC)

## Abstract

**Background:**

Remote ischemic preconditioning (RIPC) protects the heart from ischemia and reperfusion (I/R) injury. The underlying molecular mechanisms are unclear. It has been demonstrated that Connexin 43 (Cx43) is critically involved in cardioprotective interventions including classical ischemic preconditioning. In the present study we investigated the influence of RIPC on the expression patterns of Cx43 after I/R in the rat heart *in vivo*.

**Methods:**

Male Wistar rats were subjected to 35 min regional myocardial ischemia followed by 2 h reperfusion with or without 4 cycles of 5 minutes bilateral hind limb ischemia and reperfusion (RIPC), to RIPC without ischemia or underwent no intervention (Sham). Infarct size was measured by TTC staining. The myocardium was divided into area at risk (AAR) and area not at risk (non AAR). Expression of Cx43-mRNA and protein was analyzed by qPCR and Western Blot analysis, respectively. Localization of Cx43 was visualized by confocal immunofluorescence staining.

**Results:**

RIPC reduced the infarct size (I/R: 73 ± 5% vs. RIPC I/R: 34 ± 14%, p < 0.05). Expression of Cx43 mRNA did not differ between groups. I/R caused a strong decrease of relative Cx43 protein expression in the AAR that was partly abolished by RIPC. Furthermore, RIPC decreased the level of ischemia-induced dephosphorylation of Cx43. Confocal immunofluorescence staining showed that I/R caused a loss of the Cx43 signal at the intercalated discs, while the Cx43 signal at the intercalated discs was partly sustained after RIPC.

**Conclusion:**

Preservation of Cx43 protein expression and phosphorylation after RIPC might protect the rat heart *in vivo*.

**Electronic supplementary material:**

The online version of this article (doi:10.1186/s12967-014-0228-8) contains supplementary material, which is available to authorized users.

## Background

In 1993 Przyklenk and colleagues published the observation that regional mycardial ischemia can protect the remote myocardial tissue from subsequent ischemia [1]. This concept – termed remote ischemic preconditioning (RIPC) – has later been extended by studies showing that a protective myocardial effect could also be achieved by short intervals of limb ischemia [2], a fact which makes RIPC easily applicable and clinically interesting. Recently, the potential clinical impact of RIPC has been demonstrated in two studies by Botker et al. [3] and Thielmann et al. [4]. Botker and colleagues demonstrated that remote ischemic conditioning induced by intermittend periods of arm ischemia before angioplasty increased myocardial salvage in patients suffering from acute myocardial infarction [3]. The clinical investigation by Thielmann and colleagues showed that RIPC by transient ischemia of the left upper arm can provide perioperative myocardial protection and improve the prognosis of patients undergoing elective coronary artery bypass surgery [4]. However, the exact molecular mechanisms underlying RIPC are unknown and elucidation of these mechanisms is of great clinical importance. One of the molecules involved in the events following cardiac ischemic preconditioning (IPC) is Cx43 [5]. Cx43 is the main gap junction component of the heart ventricle and is mainly located at the intercalated discs. It contributes to mechanical stability as well as electrical and chemical coupling of cardiomyocytes [6]. After prolonged ischemia, Cx43 is dephosphorylated [7] which leads to the opening of Cx43-formed hemichannels [8] and a translocation of Cx43 from the gap junctions towards the plasma membrane [9] or to an intracellular pool [10]. IPC attenuates this ischemia-induced dephosphorylation of sarcolemmal Cx43 in rat hearts [11]. Furthermore, heterozygous deficiency of Cx43 in mice leads to a loss of the cardioprotection by IPC [12,13].

There is no study showing an impact of RIPC on the Cx43 phosphorylation status or Cx43 protein distribution in the context of cardiac I/R. We hypothesised that RIPC preserves Cx43 phosphorylation and therefore analyzed the expression pattern of Cx43 in a rat model of RIPC followed by I/R.

## Methods

### Animal experiments

Animal experiments were performed in accordance with the German legislation on protection of animals and the National Institutes of Health Guide for the Care and Use of Laboratory Animals (NIH Publication No. 85–23, revised 1996) as described previously [14]. Animals had free access to food and water at any time. Rats were anesthetized by an intraperitoneal injection of pentobarbital (100 mg/kg body weight). Before surgery, adequate depth of anesthesia was verified by the absence of reaction after pain stimuli. After tracheal intubation, the right jugular vein was cannulated for saline and drug infusion, and the left carotid artery was cannulated for measurement of aortic pressure. A lateral left-sided thoracotomy was performed and a suture (5–0) was looped around a major branch of the left coronary artery. All animals were left untreated for 10 min before the start of the respective experimental protocol.

### Experimental *in vivo* protocol

**Figure 1:**
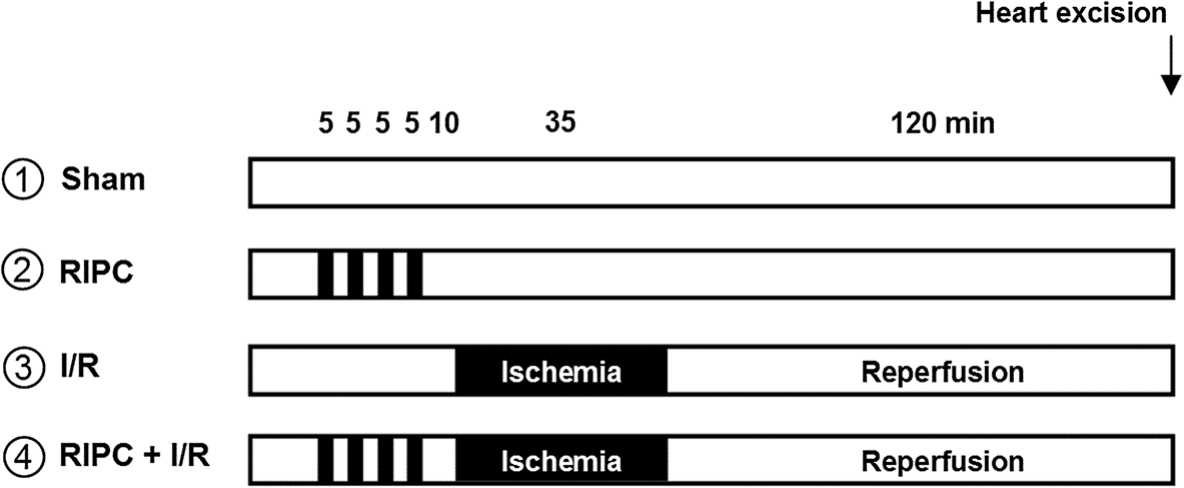
**Experimental** ***in vivo*** **protocol. RIPC = remote ischemic preconditioning, I/R ischemia and reperfusion, n = 6 / group.**

Myocardial ischemia and RIPC were performed as described before. [14]. In brief, rats were randomly assigned to one of the following groups (Figure 1, n = 6 / group): 1. Sham: Animals underwent a time matched sham procedure without intervention. 2. Remote ischemic preconditioning only (RIPC): 4 cycles of 5 minutes bilateral hind limb ischemia/5 minutes reperfusion without I/R. 3. Ischemia/Reperfuion (I/R): 35 minutes of regional myocardial ischemia by occlusion of a branch of the left coronary artery (LAD) followed by 120 minutes of reperfusion. 4. Remote ischemic preconditioning followed by ischemia/reperfusion (RIPC + I/R). At the end of the experiment, the branch of the LAD was re-occluded and 5 ml Evans blue solution were injected intravenously. By this method, the area non at risk (non AAR) is stained blue while the area at risk (AAR) remains unstained. Subsequently, the hearts were removed, and the myocardium was separated in AAR and nonAAR. Both tissue fractions were snap frozen in liquid nitrogen and stored at −80°C until further analysis.

In a second series, the same experimental protocol was used to assess infarct size in I/R and RIPC + I/R animals (n = 6 / group).

### Infarct size measurement

Infarct size measurement was performed as described previously [15]. In brief, after 120 min of reperfusion, the hearts were excised with the occluding suture left in place and then mounted on a modified Langendorff apparatus for perfusion with ice cold normal saline. After 5 min of perfusion, the coronary artery was re-occluded and the heart perfused with 0.2% Evans blue in normal saline for 10 min. Intravascular Evans blue was washed out by perfusion with normal saline for 10 min. This treatment identified the area at risk as unstained. The heart was cut into 2 mm thick transverse slices. The slices were stained with 0.75% triphenyltetrazolium chloride solution for 15 min at 37°C and fixed in 4% formalin solution for 24 h at room temperature. The area at risk and the infarcted area were determined by planimetry using SigmaScan Pro 5® computer software (SPSS Science Software, Chicago, IL).

### RNA isolation

Total RNA of rat hearts was isolated using Trizol reagent (Invitrogen, Carlsbad, USA) according to the manufacturer’s protocol. RNA quantity was determined by UV spectrophotometry (Nanodrop, Thermo Scientific, USA) and RNA integrity was verified by agarose gel electrophoresis using 2.5 μg of total RNA per lane.

### RNA-qPCR assay

1 μg of total RNA was reverse transcribed using the High Capacity RNA-to-cDNA Master Mix according to the manufacturer’s protocol (Applied Biosystems). The qPCR assay for Cx43 was generated by TIB MOLBIOL (Berlin, Germany). The sequence of the forward primer is 5′-AGGAGTTCCACCAACTTTGGC-3′, reverse primer 5′-TGGAGTAGGCTTGGACCTTGTC-3′ and 5′-FMA-AGCTTCCCCAAGGCACTCCAGTC-BBQ-3′ for the reporter probe. GAPDH (Assay ID: Rn_01775763, Applied Biosystems) was used for normalization. qPCR conditions: 50°C for 2 min, 95°C for 10 min, 40 cycles of 95°C for 15 s, 60°C for 60 s on an Applied Biosystems 7300HT thermocycler (Applied Biosystems). All samples were run in triplicates and PCR was repeated twice. Relative expression was estimated using the ΔΔCq-method [16] and the relative expression software tool [17].

### Subcellular fractionation

The membrane fraction of proteins was obtained by differential centrifugation. The frozen heart tissue was pulverized and dissolved in lysis buffer containing 5 mM Tris base, 2 mM EGTA, 50 mM NaF and 2 mM Na_3_VO_4_ (as phosphatase inhibitors), a freshly added protease inhibitor mix (Complete; Roche) and 5 mM DTT. The solution was vigorously homogenized on ice (Homogenizor; IKA, Staufen, Germany) and then centrifuged at 600 g at 4°C for 10 min. The supernatant was centrifuged at 15.000 g at 4°C for 15 minutes, followed by ultracentrifugation at 100.000 g at 4°C for 1 h. The pellet was resuspended with lysis buffer containing 1% Triton and incubated on ice for 60 min. The supernatant containing the membrane fraction was transferred to a new tube for further analysis.

### Western blotting

Protein concentration was measured by the Lowry method and equal amounts of protein were mixed with loading buffer (1:1) containing Tris–HCl, glycerol, sodium dodecyl sulfate and bromphenol blue. Samples were mixed 1:10 with 2-β-mercaptoethanol and incubated at 95°C for 5 min, and loaded on a 10% SDS-polyacrylamide gel. The proteins were separated by electrophoresis and transferred onto a polyvinylidene difluoride membrane by tank blotting (100 V, 1 h). Unspecific binding of the antibody was blocked by incubation with 5% nonfat dry milk in Tris-buffered saline containing Tween 20 for 2 h. The membrane was incubated overnight at 4°C with the primary antibody (Cx43, ab11370, abcam, Cambridge, UK, 1:1000). After washing in fresh, cold Tris-buffered saline containing Tween, the blot was incubated with the appropriate horseradish peroxidase conjugated secondary antibody for 2 h at room temperature. Immunoreactive bands were visualized by chemiluminescence detected with a high-resolution camera using an enhanced chemiluminescence system (Santa Cruz Biotechnology, Santa Cruz, Calif). The signal intensities of the corresponding bands in Western blot were measured using GelScan 6.0 software (Decon Science Tec, Frankfurt, Germany). Equal loading of protein was verified by probing the membrane with Na^+^/K^+^-ATPase antibody (Abcam ab 7671, Cambridge, UK, 1:5000).

### Immunofluorescence staining and confocal laser scanning microscopy

Frozen heart tissue was cut into 8 μm-thick sections, fixed in 4% paraformaldehyde for 10 min at room temperature and washed with phosphate buffered saline. Subsequently, cryosections were blocked and permeabilized for 1 h with blocking buffer containing 10% normal goat serum and 0.2% saponine in phosphate buffered saline.

Subsequently, cryosections were incubated with the primary Cx43 antibody (Abcam ab11370, Cambridge, UK, 1:1000) at 4°C overnight. A secondary antibody (Cy3-conjugated goat-anti-rabbit, Jackson Immuno Research Laboratories, USA, Code 111-165-144, 1:400) was applied for 4 h at room temperature. Glass slides were washed with phosphate buffered saline and mounted to coverslips with mounting medium (ProLong Gold antifade reagent with DAPI; Invitrogen). Immunostained samples were analyzed using a Zeiss LSM510META confocal microscope.

### Statistical analyis

All values were tested for normal distribution and are expressed as mean ± SD. Hemodynamic data were analyzed by two-way analysis of variance (ANOVA) followed by Tukey’s post hoc test (Sigmastat Software, version 3.5). After failing testing for normal distribution, a Kruskal-Wallis test followed by Dunn’s posthoc testing was performed for analyzing Western Blot data of Cx43 protein expression. P < 0.05 was used to indicate statistical significance.

## Results

### Hemodynamic variables

No significant differences in hemodynamic baseline variables were observed between treatment groups. Heart rate was significantly lower in the I/R and the RIPC + I/R group after 120 minutes of reperfusion compared to baseline values and to Sham animals. Blood pressure was significantly lower in I/R and RIPC + I/R animals after 120 minutes of reperfusion compared to baseline values and was lower in the I/R group compared to Sham animals (details in Additional file 1: Table S1 and S2).

### Infarct size measurement

**Figure 2:**
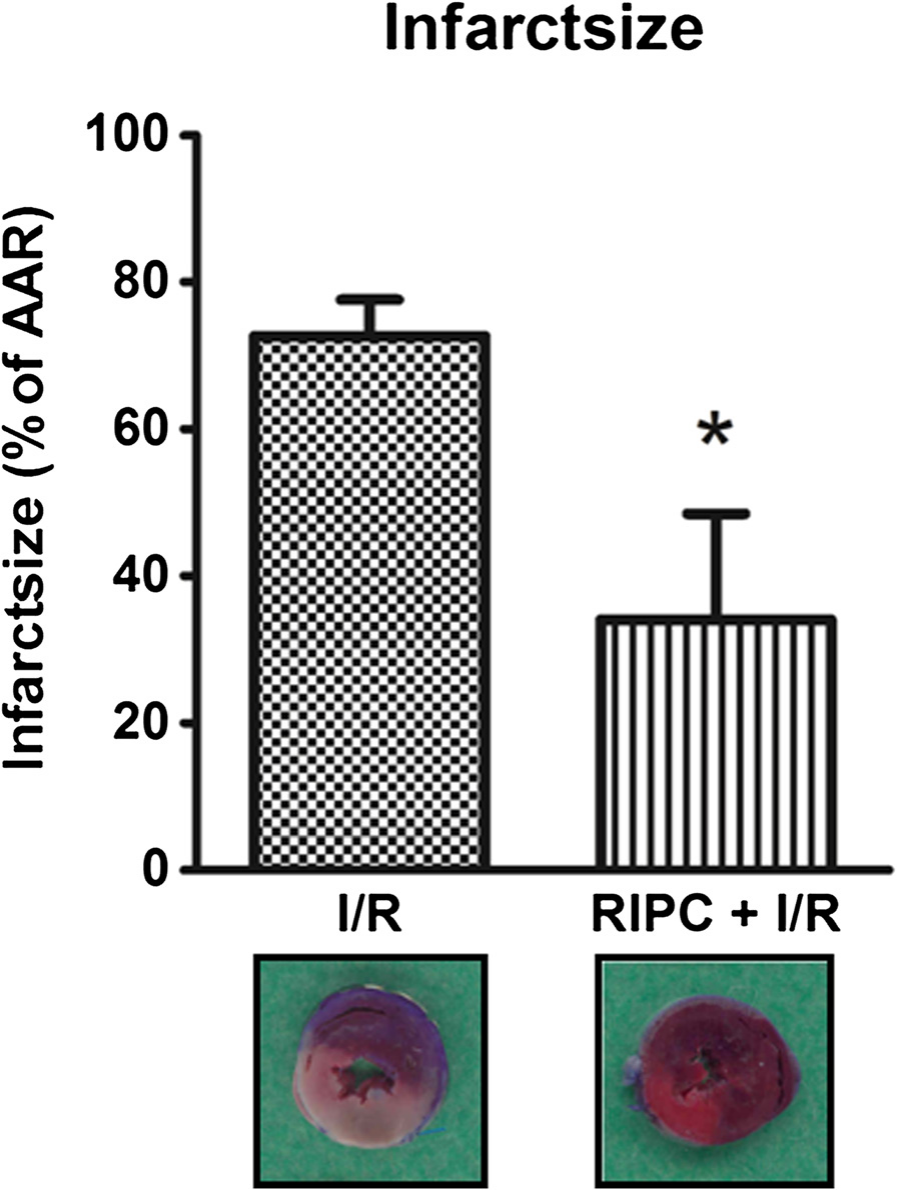
**RIPC leads to a significant decrease of the infarct size after I/R in percentage of the area at risk.** RIPC = remote ischemic preconditioning, I/R ischemia and reperfusion (Data are mean ± SD, n = 6 / group. * p < 0.05). Representative pictures of TTC-stained hearts from I/R and RIPC-I/R animals are shown (white: infarct area, red: AAR, blue: area not at risk, not perfused by LAD).

In the I/R group, infarct size was 72.6 (±5) % (Figure 2). Infarct size was reduced by RIPC to 34.1 (±14) % (p < 0.01, n = 6). Furthermore, there was no difference in the area at risk between groups (data not shown), therefore the differences in infarct sizes were not caused by differences in area at risk.

### Cx43 mRNA expression

**Figure 3:**
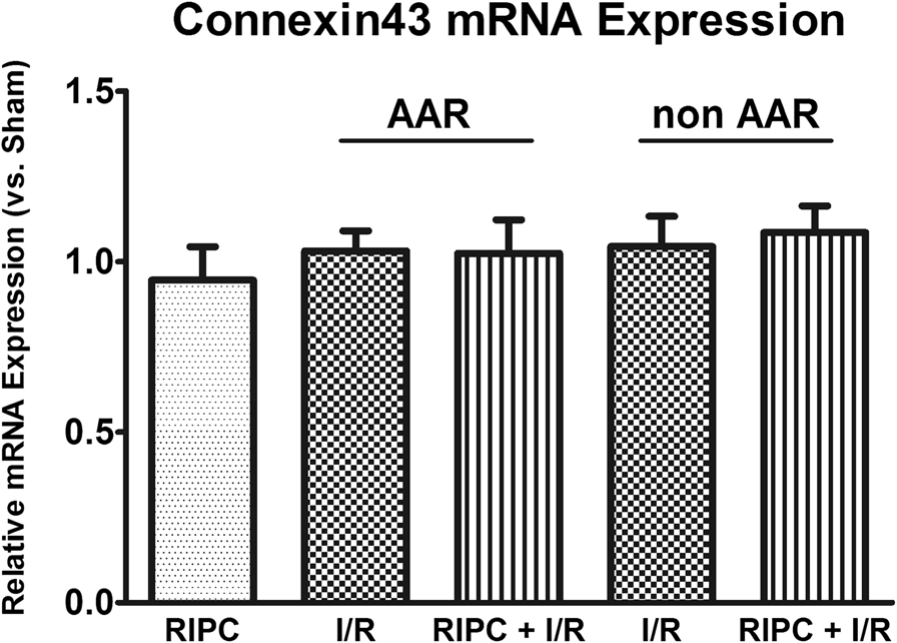
**Relative Cx43 mRNA expression measured by qPCR.** There is no differential mRNA expression between the groups (Data are mean ± SD, n = 6 / group).

Realtime quantitative PCR was performed to analyze the expression of Cx43 mRNA. There were no differences in the expression level of Cx43 mRNA between the groups (Figure 3).

### Cx43 protein expression

Cx43 protein expression was analyzed by Western Blotting using the membrane fractions of heart tissue. A Cx43 antibody detecting both phosphorylated as well as unphosphorylated Cx43 was applied.

**Figure 4:**
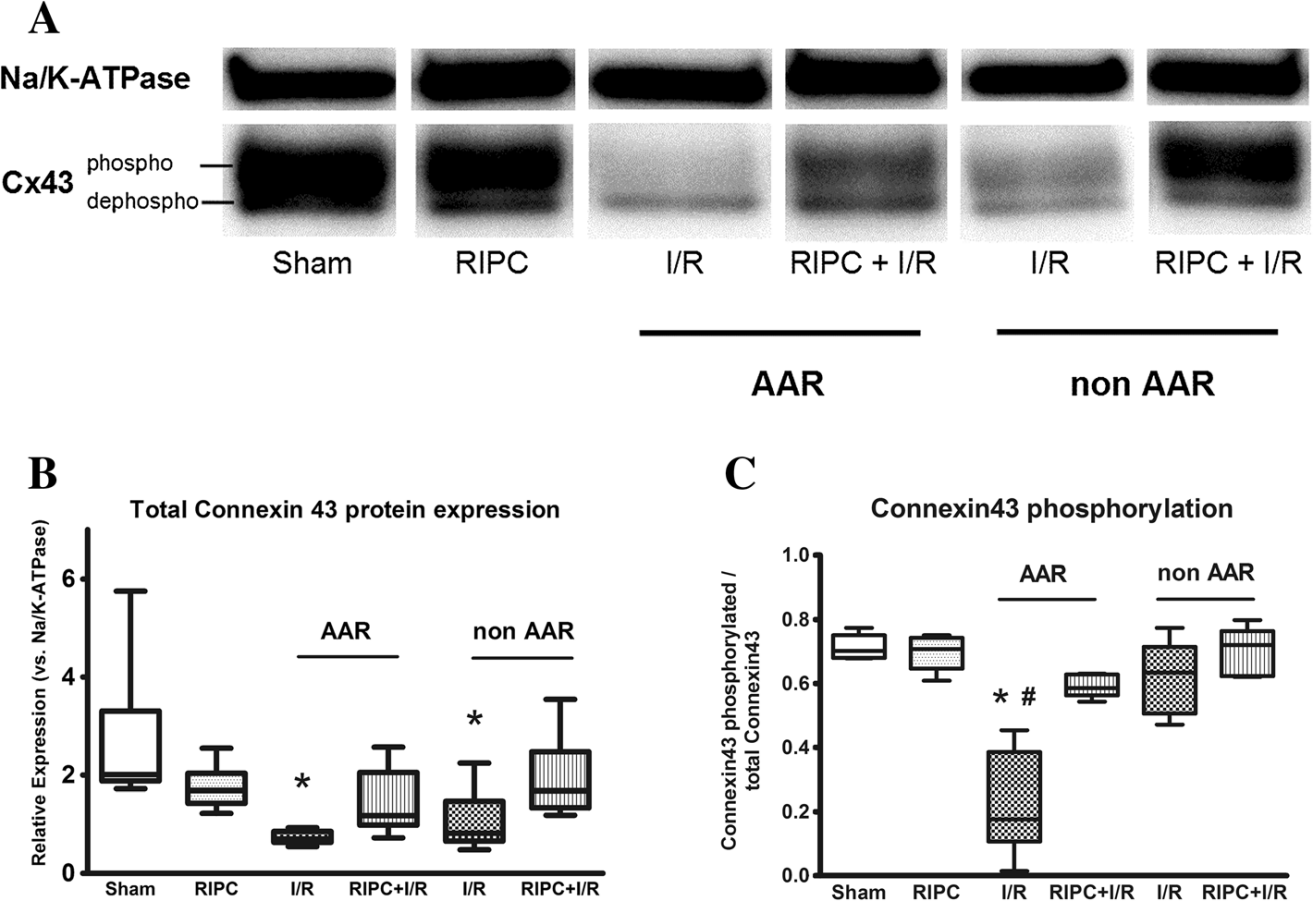
**Western Blot analysis of Cx43 protein expression. (A)** Exemplary original blot showing the expression of Cx43 and Na^+^/K^+^-ATPase. The upper Cx43 band represents the expression of phosphorylated Cx43, while the lower band corresponds to dephosphorylated Cx43. **(B)** Relative expression of total Cx43 protein vs. Na^+^/K^+^-ATPase. Cx43 expression is significantly lower in the AAR of I/R hearts compared to Sham. **(C)** Analysis of phosphorylated vs. dephosphorylated Cx43. The degree of dephosphorylated Cx43 is higher in the AAR of I/R hearts compared to Sham and RIPC + I/R hearts. RIPC = remote ischemic preconditioning, I/R = ischemia and reperfusion, (Data are median, min to max, n = 6 / group. * p < 0.05).

Total Cx43 protein expression was significantly reduced in the AAR of hearts subjected to I/R hearts compared to Sham hearts (relative expression versus Na^+^/K^+^-ATPase: 0.73 for I/R vs. 2.65 for Sham, p < 0.05) (Figure 4b). Interestingly, this decrease is not only observed in the AAR but also in the non AAR of I/R hearts (relative expression 1.05 versus Na^+^/K^+^-ATPase, p < 0.05). In contrast, there is no significant decrease in Cx43 protein expression in the AAR of hearts pretreated with RIPC (RIPC + I/R, relative expression 1.43).

Cx43 can be phosphorylated at several sites including Ser^365^, Ser^325^, Ser^328^ and Ser^330^ and phosphorylation of these sites is known to affect SDS/PAGE migration [18]. The fastest migrating band is termed P0, it is the dephosphorylated form of Cx43. For analyzing the phosphorylation status of Cx43, we determined the ratio of phosphorylated to total Cx43 protein content. Phosphorylation of Cx43 was significantly decreased in the AAR of I/R hearts (Phosphorylated Cx43/total Cx43 = 0.22) in comparison to Sham (0.71) as well as in comparison to the AAR of RIPC + I/R hearts (0.59) (Figure 4c). Thus, RIPC prevents the I/R-induced dephosphorylation of Cx43.

### Immunofluorescence staining of Cx43

**Figure 5:**
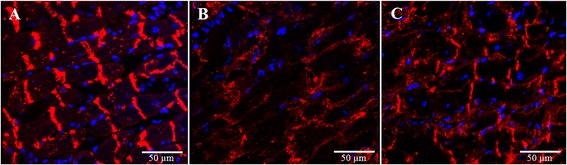
**Immunofluorescence staining of Cx43 in rat hearts. (A)** Cx43 is mainly located at the intercalated discs of Sham hearts. **(B)** In I/R hearts, the Cx43 signal at the intercalated discs is lost and Cx43 is partly lateralized to the plasma membrane. **(C)** RIPC leads to a partial preservation of Cx43 at the intercalated discs after I/R. RIPC = remote ischemic preconditioning, I/R = ischemia and reperfusion.

To analyze the distribution of Cx43, heart tissue was labelled with a Cx43 antibody. In Sham animals, the strongest Cx43 signal was detected at the intercalated discs (Figure 5A). In contrast, in the AAR of I/R sections the Cx43 signal at the intercalated discs was lost and Cx43 could to a lesser degree be detected in the cardiomyocyte plasma membrane (Figure 5B). In the AAR of heart sections treated with RIPC before I/R, some of the Cx43 protein content at the intercalated discs was preserved and to a lesser amount lateralized to the plasma membrane (Figure 5C).

## Discussion

In this study we show that RIPC 1) attenuates the ischemia-induced dephosphorylation of Cx43, 2) partially prevents the lateralization of Cx43 from the intercalated discs to the plasma membrane, and 3) reduces the degree of Cx43 degradation.

Though being first described 20 years ago [1], the exact molecular mechanisms underlying RIPC are unknown. Humoral factors, a neural pathways or microRNAs [19] have been proposed for mediating the cardioprotective effects following RIPC (reviewed in [20]). The molecular events following IPC, where myocardial I/R is induced by direct transient ligation of a coronary artery, are better characterized than the mechanisms underlying RIPC, where the ischemic event occurs in an organ distinct from the heart. While some studies suggest that the molecular events mediating RIPC are similar to events following IPC [21], a study of our group has shown that RIPC exhibits a different signalling pattern compared to IPC [14]. One protein that has been extensively studied in the context of IPC is Cx43. Cx43 is an integral membrane protein that is mainly localized at the sarcolemma of ventricular cardiomyocytes where six connexin molecules assemble into a connexon or hemichannel [22]. The protein can be phosphorylated at numerous Serin sites including Ser^325^, Ser^328^, Ser^330^, Ser^365^ and Ser^262^. Phosphorylation (or dephosphorylation respectively) is able to induce conformational changes within the Cx43 molecule [23] and the phosphorylation status of Cx43 is related to stress response and resistance of cells to injury [24]. Cx43 is a target protein of several protein kinases such as protein kinase A (PKA), protein kinase C (PKC), protein kinase G (PKG), protein tyrosine kinases and mitogen activated protein kinases (MAP kinases) [7].

### Myocardial ischemia, Cx43 and cardioprotection

It has been shown that prolonged myocardial ischemia leads to a dephosphorylation of Cx43 [25]. Our data confirm this finding. In addition, myocardial ischemia causes a re-distribution of Cx43 to the lateral plasma membrane. Besides this lateralization, some of the Cx43 is degraded following prolonged cardiac ischemia [26].

In our experimental model, we could reproduce both findings, lateralization and degradation of Cx43 caused by myocardial I/R. These effects of myocardial I/R on Cx43 dephosphorylation, lateralization and degradation can be modulated by cardioprotective interventions including IPC. Ischemia-induced dephosphorylation of sarcolemmal Cx43 is attenuated by IPC in rat hearts [11], which may be related to an enhanced association of Cx43 with kinases such as PKC and p38 mitogen-activated protein kinases [27]. Furthermore, heterozygous deficiency of Cx43 in mice leads to a loss of cardioprotection by IPC [12,13]. There are no data dealing with the role of Cx43 in RIPC. We therefore analyzed a possible impact of RIPC on both Cx43 mRNA and protein expression levels and cellular distribution in the context of cardiac I/R in male Wistar rats. Our data demonstrate that RIPC protects against the I/R-induced dephosphorylation of Cx43. RIPC also attenuates the I/R-induced degradation and lateralization of Cx43. Dephosphorylation of Cx43 increases the permeability of gap junctions and thereby contributes to the propagation of I/R injury [28], as during reperfusion following prolonged ischemia a death factor (such as sodium ions) is able of spreading more easily between cells [29]. An uncoupling of this propagation as a consequence of a preservation of phosphorylation is potentially cardioprotective. Therefore, presevation of Cx43 phosphorylation and protein content as shown in our study could contribute to the protective effects of RIPC. We furthermore show that the preservation of Cx43 protein is not associated with changes in Cx43 mRNA expression. This is in line with other studies showing that changes in Cx43 protein content and phosphorylation following hypoxia are not accompanied by changes in Cx43 mRNA expression [30].

In IPC, the preserved phosphorylation of Cx43 is related to an enhanced association of Cx43 with kinases such as PKC and p38 mitogen-activated protein kinases (MAPK) [27], while it is independent on the RISK and SAFE pathway [31]. We could show previously that the PKC-MAPK pathway is most likely not involved in the protective effects of RIPC [14]. It therefore remains to be elucidated how RIPC contributes to the preservation of Cx43 phosphorylation on a molecular level.

While most Cx43 molecules are found in the sarcomlemma of cells, Cx43 is also located in the mitochondrial membrane of mammalian cardiomyocytes [32,33]. It has been shown that mitochondrial Cx43 is involved in the events following IPC [32], but there are no data dealing with mitochondrial Cx43 in the context of RIPC. Our study however focused on sarcolemmal Cx43 and further studies are necessary to analyze a potential influence of mitochondrial Cx43 in the context of RIPC.

## Conclusion

In summary, our data show that RIPC has an impact on ischemia-induced Cx43 dephosphorylation, lateralization and degradation. Further studies are necessary to investigate the exact molecular events leading to the preserved phosphorylation of Cx43 following RIPC and a potential impact of mitochondrial Cx43.

## Additional file

Additional file 1: Table S1. Hemodynamic variables molecular biology experiments. **Table S2.** Hemodynamic variables infarct size series.

## Abbreviations

AAR: Area at risk
Cx43: Cx43
IPC: Ischemic preconditioning
I/R: Ischemia and reperfusion
nAAR: Area not at risk
RIPC: Remote ischemic preconditioning
