# Supplementary material for: Remote ischemic preconditioning preserves Connexin 43 phosphorylation in the rat heart in vivo
Source: J Transl Med. 2014 Aug 27;12:228. doi: 10.1186/s12967-014-0228-8 (PMC4256705; doi:10.1186/s12967-014-0228-8)
Supplement: Additional file 1: Table S1. — Hemodynamic variables molecular biology experiments. Table S2. Hemodynamic variables infarct size series. [file 12967_2014_228_MOESM1_ESM.doc]

**Additional file 1: Table S1:** Hemodynamic variables molecular biology experiments

|  | Baseline | | |  | Ischemia | | |  | Reperfusion | | | | | |
| --- | --- | --- | --- | --- | --- | --- | --- | --- | --- | --- | --- | --- | --- | --- |
|  |  | | |  | 15 min | | |  | 30 min | | | 120 min | | |
| *Heart Rate (bpm)* |  | | | | | | | | | | | | | |
| Sham | 407 | ± | 47 |  | 395 | ± | 45 |  | 389 | ± | 46 | 382 | ± | 55 |
| RIPC | 403 | ± | 19 |  | 372 | ± | 28 |  | 381 | ± | 42 | 337* | ± | 40 |
| RIPC + I/R | 406 | ± | 28 |  | 385 | ± | 21 |  | 343* | ± | 19 | 332*^#^ | ± | 23 |
| I/R | 391 | ± | 37 |  | 389 | ± | 23 |  | 333^#^ | ± | 9 | 315*^#^ | ± | 22 |
|  | | | | | | | | | | | | | | |
| *Mean AOP (mmHg)* |  | | | | | | | | | | | | | |
| Sham | 124 | ± | 23 |  | 108 | ± | 16 |  | 107 | ± | 15 | 99 | ± | 11 |
| RIPC | 133 | ± | 15 |  | 113 | ± | 24 |  | 103* | ± | 20 | 87* | ± | 11 |
| RIPC + I/R | 129 | ± | 15 |  | 125 | ± | 9 |  | 93* | ± | 15 | 86* | ± | 11 |
| I/R | 133 | ± | 11 |  | 131^#^ | ± | 13 |  | 90* | ± | 12 | 69*^#^ | ± | 10 |

Data are mean ± SD. * p < 0.05 vs. baseline ^#^ p < 0.05 vs. Sham

RIPC = remote ischemic preconditioning, I/R ischemia and reperfusion, n=6 / group.

**Additional file 1: Table S2:** Hemodynamic variables infarct size series

|  | Baseline | | |  | Ischemia | | |  | Reperfusion | | | | | |
| --- | --- | --- | --- | --- | --- | --- | --- | --- | --- | --- | --- | --- | --- | --- |
|  |  | | |  | 15 min | | |  | 30 min | | | 120 min | | |
| *Heart Rate (bpm)* |  | | | | | | | | | | | | | |
| RIPC + I/R | 387 | ± | 18 |  | 383 | ± | 52 |  | 367 | ± | 37 | 358 | ± | 50 |
| I/R | 380 | ± | 15 |  | 386 | ± | 26 |  | 375 | ± | 30 | 373 | ± | 35 |
|  | | | | | | | | | | | | | | |
| *Mean AOP (mmHg)* |  | | | | | | | | | | | | | |
| RIPC + I/R | 121 | ± | 12 |  | 117 | ± | 19 |  | 113 | ± | 16 | 98 | ± | 22 |
| I/R | 115 | ± | 20 |  | 107 | ± | 20 |  | 95 | ± | 21 | 83 | ± | 18 |

Data are mean ± SD.

RIPC = remote ischemic preconditioning, I/R ischemia and reperfusion, n=6 / group.
